# Hippocampal Neuroligin-2 Overexpression Leads to Reduced Aggression and Inhibited Novelty Reactivity in Rats

**DOI:** 10.1371/journal.pone.0056871

**Published:** 2013-02-22

**Authors:** Christine Kohl, Orbicia Riccio, Jocelyn Grosse, Olivia Zanoletti, Céline Fournier, Mathias V. Schmidt, Carmen Sandi

**Affiliations:** 1 Laboratory of Behavioral Genetics, Brain Mind Institute, School of Life Sciences, Ecole Polytechnique Fédérale de Lausanne, Lausanne, Switzerland; 2 Max Planck Institute of Psychiatry, Munich, Germany; Sapienza University of Rome, Italy

## Abstract

Disturbances of the excitation/inhibition (E/I) balance in the brain were recently suggested as potential factors underlying disorders like autism and schizophrenia resulting in associated behavioral alterations including changes in social and emotional behavior as well as abnormal aggression. Neuronal cell adhesion molecules (nCAMs) and mutations in these genes were found to be strongly implicated in the pathophysiology of these disorders. Neuroligin2 (nlgn2) is a postsynaptic cell adhesion molecule, which is predominantly expressed at inhibitory synapses and required for synapse specification and stabilization. Changes in the expression of nlgn2 were shown to result in alterations of social behavior as well as altered inhibitory synaptic transmission, hence modifying the E/I balance. In our study, we focused on the role of nlgn2 in the dorsal hippocampus in the regulation of emotional and social behaviors. To this purpose, we injected an AAV construct overexpressing nlgn2 in the hippocampus of rats and investigated the effects on behavior and on markers for the E/I ratio. We could show an increase in GAD65, a GABA-synthesizing protein in neuronal terminals, and furthermore, reduced exploration of novel stimuli and less offensive behavior. Our data suggest nlgn2 in the hippocampus to be strongly implicated in maintaining the E/I balance in the brain and thereby modulating social and emotional behavior.

## Introduction

Cell adhesion molecules are crucially involved in synapse development and stabilization [1], [2], [3]. Neuroligins are postsynaptic neuronal cell adhesion molecules, which link the presynapse to the postsynaptic density by binding to their presynaptic partners neurexins in an alternative splice-dependent manner [4]. They were initially thought to be required for synapse formation, but recent studies indicated a crucial involvement rather in synapse maturation and specification [5]. Four genes encode for the different members of the neuroligin (nlgn) family in mammals, nlgn 1–4, which are differentially enriched in postsynaptic specializations of synapses. Nlgn1 and nlgn2 localize in excitatory and inhibitory synapses, respectively, and nlgn3 seems to be present in both [6], [7], [8]. Consistent with this, knockout of nlgn1 was shown to impair NMDA-receptor mediated signaling, whereas nlgn2-KO was reported to result in deficits in inhibitory synaptic transmission [9], [2]. Due to their differential expression in different types of synapses, neuroligins were implicated in the control of excitatory versus inhibitory synapse specification and herewith the regulation of the excitation/inhibition (E/I) balance in the hippocampus [10].

Emerging evidence implicates neuroligins in autism spectrum disorders (ASD) and schizophrenia, neurodevelopmental conditions characterized, among other symptoms, by abnormal or impaired development in social interaction and communication and a markedly restricted repertoire of activity and interest [11], [12]. A disturbance of the E/I balance in the brain was put into the spotlight as important potential factor underlying these disorders [9], [13], [14], [15], [16]. Lately, genome-wide copy number variation studies identified mutations in different neuroligins (nlgn1, nlgn3 and nlgn4) and their molecular partners in these disorders [17], [18], [19]. Furthermore, mutations in the genes encoding nlgn3 and nlgn4 were implicated in the pathophysiology of autism [20], [21], [9], [22], [23] and nlgn2 was shown to be associated with schizophrenia [24] and certain symptoms of autism in mice [25]. Nlgn2 has also been proposed as a potential candidate gene for mental retardation or ASD [26].

Nlgn2 expression has been shown to be altered in the hippocampus under a number of genetic [27] and life [28], [29] conditions associated with changes in social behaviors. For example, transgenic mice overexpressing nlgn2 under the Thy1 promotor showed stereotyped jumping, anxiety-like behavior as well as impairments in social interactions [25]. Although in this mouse model, nlgn2-OE was confirmed in cortex and limbic structures, such as amygdala and hippocampus, most attention was focused on the prefrontal cortex (PFC) that was confirmed to display overall reduction in the E/I ratio. In line with those findings, recent evidence has validated the importance of the E/I balance in the PFC in modulating social behavior [30]. However, the possibility that alterations in nlgn2 content in other brain regions, such as the hippocampus, are involved in relevant behavioral alterations has to been explored. Besides its implications in cognitive function, the hippocampus represents an important candidate for regulating contextually appropriate emotional behavior [31] and abnormalities in this brain structure have been implicated in pathological aggression and autism [32], [33], [34]. Specifically, the dorsal hippocampus has been shown to mediate anxiogenic effects in the social interaction test [35].

In the present study, we focused on the role of nlgn2 in the adult dorsal hippocampus and asked whether a viral overexpression of nlgn2 in the adult dorsal hippocampus of rats leads to alterations in emotional and social behaviors. With this approach, we aimed at directly addressing the role if this molecule when manipulated at adulthood, avoiding possible developmental effects that can be expressed in nlgn2-OE animals in which overexpression is already present during development.

## Material and Methods

### Animals

All experimental animals were the male offspring of Wistar rats obtained from (Charles River Laboratories, France). After birth, all animals stayed with the dam until weaning on postnatal day 21. Male rats from different litters were housed in groups of three under standard conditions in plastic standard cages (42×28×20 cm) with a 12 h light-dark cycle (lights on at 7∶30 AM) and controlled temperature and humidity (22±2°C; 50±20%). Food and water were available *ad libitum*. All procedures described were conducted according to the Swiss National Institutional Guidelines of Animal Experimentation and were approved through a license issued by the Cantonal Veterinary Authorities (Vaud, Switzerland). All efforts were made to minimize animal suffering during the experiments.

### Experimental Design

After assessing anxiety levels at the age of 11 weeks, animals were equally distributed to two groups with comparable anxiety-like behavior and then subjected to surgery with injection of an empty vector (n = 10) or a nlgn2-overexpressing construct (nlgn2-OE, n = 10). Following a recovery period of ca. 5 weeks, animals underwent behavioral testing. All behavioral tests were performed between 08∶00 AM and 14∶00PM, except of the resident-intruder test which took place in the dark phase, starting from 07∶30 PM. We measured the body weight of all animals in a weekly fashion throughout the experiment, beginning on the day of surgery for the respective virus injection. A schematic overview of the experiment is depicted in supplemental figure S1. In all behavioral tests, video-recording (MediaCruise, Canopus Co., Ltd.) and an automated-tracking system (Ethovision 3.1, Noldus IT) was used.

### Viral Overexpression of nlgn2

At the age of 12 weeks, animals were subjected to surgeries for the viral overexpression of nlgn2 in the dorsal hippocampus, wherein an adeno-associated AAV1/2 vector (http://www.genedetect.com) containing a CAG-HA-tagged-nlgn2-WPRE-BGH-polyA- expression cassette was used. Control animals were injected with an empty construct (AAV1/2-CAG-Null/Empty-WPRE-BGH-polyA). All viral constructs used were designed and produced by GeneDetect, New Zealand. The vector incorporated the following regulatory elements: rAAV2 inverted terminal repeat (ITR) sequences, a scaffold attachment region (SAR) element, the hybrid chicken B‐actin/CMV enhancer (CAG) promoter region, a cis‐acting woodchuck post‐transcriptional regulatory element (WPRE) and a bovine growth hormone polyadenylation signal sequence (bgh‐polyA). The mouse pCAG-HA-tagged-Nlgn2 plasmid was kindly provided by Prof. Dr. Peter Scheiffele, University of Basel, Switzerland. For the empty vector, the same backbone without the cDNA was used.

Initially, animals were anaesthetized with an intraperitoneal injection of ketamine (100 mg/kg body weight) and xylazine (10 mg/kg body weight) and installed in a stereotactic frame to avoid any head movements during the surgical procedure. A total of 2 µl of either nlgn2-OE or empty vector (titres: Nlgn2-OE: 1.4×10^12^ genomic particles/ml; empty: 1.3×10^12^ genomic particles/ml) was bilaterally injected (two injection sites per side, 1 µl each) in the dorsal hippocampus using automated syringe pumps with a flow rate of 0.2 µl/min. The injectors were left in site for additional 5 minutes after the actual injection. To target the dorsal area of the hippocampus following coordinates were used: 3.5 mm posterior to bregma, 1.5 mm and 3.5 mm from midline, 3.5 mm ventral from skull. After removing the injectors, animals were injected with an antisedant (0.1 mg/kg body weight, Antisedan, Pfizer). All rats were treated with paracetamol (500 mg/700 ml H_2_O, Dafalgan, Bristol-Myers Squibb, Agen, France) via the drinking water for 7 days after the surgery. The animals were allowed to recover for 5 weeks from surgery before the behavioral testing was started.

### Behavioral Analysis

#### Elevated plus maze (EPM)

To assess the animals’ anxiety-related behavior before surgery, the elevated plus maze was used. The apparatus consisted of a plus-shaped elevated platform (42 cm above the ground) with two opposing open arms (50×10 cm), two opposing closed arms (50×10×38 cm) and a central platform (10×10 cm), made of black PVC. Light conditions were set to 15–16 lux in the open arms and 5–6 Lux in the closed arms. The rats were individually placed onto the maze facing one of the closed arms and were allowed to explore the apparatus for 5 minutes. The maze was cleaned with 2% Ethanol after every run. According to the percentage of time spent in the open arms, animals were grouped for AAV surgeries.

### Open Field – Novel Object (OF-NO)

The open field-novel object test was performed to evaluate any effects of nlgn2- overexpression after surgery on the animals’ emotional and exploratory behavior. The open field test (OF) was conducted in a circular open arena (1 m in diameter, 40 cm high) by placing the animal near the wall and leave it to freely explore the apparatus for 10 minutes. The OF was virtually divided in three parts: center zone in the middle of the arena with a diameter of 25 cm, an intermediate zone with a diameter of 75 cm and the remaining wall zone along the walls of the arena. The parameters analyzed were the total distance travelled during the test, the percentage of time spent in each zone and the number of rearings.

Immediately after the OF testing, an object (white plastic bottle) was introduced to the center of the arena and each animal was given another 5 minutes to explore the whole arena including the novel object. Parameters of interest in this test were the percentage of time spent in each zone, the total distance and the number of rearings.

The arena was cleaned with 2% Ethanol after every animal.

### Sociability – Social Memory Test (SP-SM)

The arena for this test consisted of a rectangular, three-chambered box with a center compartment (20×35×35 cm) and two side compartments (30×35×35 cm) made of grey PVC. The dividing walls between the compartments were equipped with retractable doorways to allow or prevent access to the side chambers. In each side chamber was a cylinder (14 cm in diameter, 35 cm high) made of transparent plastic and supplied with holes (1.5 cm in diameter) to prevent any type of direct physical contact, but to allow olfactory contact and social approach. The light was kept at 2–3 lux with homogenous illumination in the chambers.

The test was performed 1 week after the OF-NO test. The combined version of sociability and social memory test consisted of two trials after a short habituation period. Each rat was placed in the center compartment with both doors closed to acclimate for 2 minutes. For the first test trial, the doors were opened and the animal was allowed to explore the whole apparatus for 5 minutes with the side chambers containing either an object (yellow box) or a juvenile Wistar rat (J1, age of 28±2 days) in the cylinder. After an inter-trial interval of 30 minutes, the animal was reintroduced to the arena for the second test trial of 5 minutes, in which the object of trial 1 was exchanged against another juvenile rat (J2). The location of objects and juveniles were counterbalanced for different animals. All juvenile rats were habituated to the cylinders for a minimum of 20 minutes on 2 consecutive days prior to testing. Cleaning with 2% Ethanol occurred after every trial.

All videos were scored manually with a focus on the percentage of time spent sniffing at each cylinder in each trial.

The sociability test was performed again 3 days after the RI test to assess any impact of an aggressive encounter on the animals’ sociability. Each rat was placed in the center compartment without access to the side chambers for 2 minutes until the doors were opened to allow free exploration of the whole arena for 5 minutes. This test consisted only of one trial in which the animal was given the choice between an object and an unfamiliar juvenile rat, which were enclosed in the cylinders in the side compartments. The locations of object and the juvenile were counterbalanced for different animals. All juvenile rats had been habituated to the cylinders for a minimum of 20 minutes on 2 consecutive days prior to the sociability -social memory testing. The arena was cleaned with 2% Ethanol after every test.

All videos were scored manually and the parameters analyzed were the percentage of time spent sniffing at the cylinders containing the object and the juvenile.

### Resident-intruder Test (RI)

Two days after SM-SP testing, all animals were separated and housed with a female. The resident-intruder test was performed on the 10th day of cohabitation. On the testing day, the female was removed from the cage 30 minutes before the start of the test (07∶30 PM) in the dark cycle. An unfamiliar Wistar rat (intruder) was introduced to the experimental animal’s home-cage for 30 minutes. All intruders were balanced for anxiety, as measured in the EPM, for every experimental group and matched for body weight to the respective resident. All videos were scored manually after the completion of the test and parameters of interest for the analysis of aggressive behavior included: the number of attacks, the latency to attack, lateral threat, keeping down, and offensive upright. The analysis for general social behavior included sniffing, mounting, grooming the intruder and self-grooming.

For the analysis of corticosterone levels following stress, blood samples were taken 0 and 30 minutes after the RI test. Therefore, a small incision in the tail of the animals was done and blood was collected in lithium heparin-coated tubes (Microvette CB 300 LH, Sarstedt AG, Nümbrecht, Germany). The samples were kept on ice and later centrifuged at 10000 rpm for 4 minutes at 4°C. Plasma was transferred to new 0.5 ml microcentrifuge tubes and stored at −20°C until further processing.

### Bedding Preference Test (BP)

As olfaction is a crucial feature for social behavior, animals were tested in the bedding preference test. Two cylinders (14 cm in diameter, 35 cm high, perforated with holes of 1.5 cm in diameter) were placed on opposite sides of the previously described OF arena. One cylinder was filled with a mixture of bedding from cages with untreated, adult male Wistar rats, whereas the other was filled with clean bedding. The animals were introduced to the arena near the wall and allowed to freely explore the whole setting for 10 minutes. The arena was cleaned with 2% Ethanol after every test. The videos were scored manually afterwards with the subsequent analysis of the percentage of time spent sniffing each cylinder.

### Sampling Procedures

Five days after the last test, 6 animals of each group were sacrificed by decapitation. Brains were removed from the skull, snap-frozen in isopentane at −40°C and stored at −80°C until further processing.

For western blots and qPCR, these brains were thawed on ice and the dorsal and ventral parts of the hippocampus were dissected. The dissected parts of the left hemisphere were further processed for qPCR, whereas the right dorsal/ventral hippocampus was utilized for western blot analysis.

The remaining four animals of each group were anesthetized with a lethal dose of pentobarbital (Esconarkon, Streuli Pharma AG, 150 mg/kg body weight, solution provided by EPFL veterinarian) and transcardially perfused with phosphate buffered saline (PBS) followed by 4% paraformaldehyde (PFA) for fixation. The brains were removed, post-fixed in 4% PFA for two days and cryoprotected in 30% sucrose/PBS for 4 days. Brain sections (30 µm) were taken in the coronal plane from the dorsal and the ventral part of the hippocampus and stored in cryoprotectant medium. A series of one in eight free-floating sections were used for immunohistochemistry for nlgn2.

### Western Blot Analyses

The samples for western blot were prepared as previously described [36]. Briefly, samples were homogenized in an homogenizer (Jencons Ltd.) containing ice-cold homogenization buffer (10 mM Hepes, 1 mM EDTA, 0.5 mM DTT, 2 mM EGTA, 0.1 mM PMSF, pH 7.5) and a Proteinase inhibitor cocktail (Roche Diagnostics GmbH, Mannheim, Germany). The homogenates were filtrated using a nylon membrane (Sefar AG, Switzerland) followed by 2 times filtration with 5 µm millipore membrane (Durapore membrane filters, Merck Millipore, Switzerland). The samples were then centrifuged and the resulting pellet was resuspended in homogenization buffer containing 1% SDS and stored at −20°C for synaptoneurosomal analysis.

All samples were quantified using the Bio-Rad DC protein assay (Bio-Rad Laboratories).

Protein samples were prepared in order to obtain equal concentrations by H_2_O dilution and mixed with 33% SDS blue loading buffer (new England Biolabs Inc, USA) containing DTT. Proteins were resolved on a 10% polyacrylamide gel and transferred onto a nitrocellulose membrane (Whatman Protran, Dassel, Germany). After blocking with 5% non-fat dry milk in PBS containing 0.2% Triton-X (PBS-T), membranes were incubated with the primary antibody/5% milk/PBS-T (nlgn2∶1:3000, Synaptic Systems, Göttingen, Germany; actin: 1∶5000, Sigma-Aldrich) overnight at 4°C. Subsequently, membranes were incubated with the secondary horseradish peroxidase-linked antibodies (for nlgn2: goat-anti rabbit, Invitrogen; for actin: goat anti-mouse, Calbiochem, USA) diluted in 5% milk/PBS-T for 1 hour. Immunocomplexes were visualized using a chemiluminescence peroxidase substrate (SuperSignal West Dura Extended Duration Substrate, ThermoScientific, USA). The immunoreactivity was detected using the ChemiDoc XRS system (Bio-Rad Laboratories). For the densitometrical analysis of the bands, Quantity One 4.6.3 software (Bio-Rad Laboratories AG, Switzerland) was used. The values are represented as adjusted volumes, normalized to actin.

### Immunohistochemistry

After incubation in 0.3% H_2_O_2_/PBS to block endogenous peroxidases, the floating sections were blocked in 10% donkey serum/PBS-T and incubated overnight with the primary antibody against nlgn2 (1∶6000, Synaptic Systems) at 4°C. Sections were rinsed in PBS-T and then incubated with the secondary antibody (biotinylated anti-rabbit IgG, 1∶2000, Vector Laboratories, USA) for 2 hours. Afterwards sections were treated using an ABC kit (Vectastain ABC Kit, Vector Laboratories, USA), followed by further washes in PBS until colour development using 3,3′-diaminobenzidine (DAB substrate kit for peroxidase, Vector laboratories).

Images were taken with the Olympus VS120-SL slide scanner using a 10× objective for whole brain images and 20× objective for hippocampus images.

### RNA Extraction and Isolation, cDNA Synthesis and Quantitative Polymerase Chain Reaction (qPCR)

Dorsal and ventral hippocampi were dissected and total RNA (tRNA) was extracted using the Trizol (Invitrogen) method. Briefly, tissues were disrupted and homogenized by using a disposable pellet mixer and cordless pestle (VWR) with 400 µl of Trizol on ice. After an incubation of 30 minutes, chloroform was added and samples were shaken vigorously. The resulting mixture was centrifuged and the supernatant was transferred into a new tube, in which isopropanol was added. Samples were then mixed, centrifuged and the resulting RNA precipitates were washed with 75% ice-cold ethanol. RNA pellets were air-dried and resuspeded in RNAse-free water.

500 ng of tRNA was converted into cDNA using the SuperScript VILO cDNA Synthesis kit (Life Technologies) according to the supplier’s recommendation.

For quantitative PCR, PCR reactions were performed in triplicates of cDNA using SYBR green PCR Master Mix (Applied Biosystems) in an ABI Prism 7900 Sequence Detection System (Applied Biosystems). Two genes were used as internal controls: 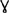-actin (actg1) and eukaryotic elongation factor-1 (eef1). Primers for the different genes of interest were designed using the Assay Design Center software from the Roche Applied Science website. Primer sequences are shown in Table 1.

**Table 1:** primer sequences for qPCR.

gene	forward primer	reverse primer
Nlgn2	5′-ccaaagtgggctgtgacc-3′	5′- ccaaaggcaatgtggtagc-3′
Nrxn1	5′-ccgagctcaggtgggttag-3′	5′-gctagactcccggatcacct-3′
Gephyrin	5′- aggagaacattctaagagccagtc-3′	5′- actgtaatgaaggctttgtccat-3′
GAD65	5′- tcttggctgtagctgacatctg-3′	5′- cgagacatcagtaaccctcca-3′
GAD67	5′- tacaacctttggctgcatgt-3′	5′- tgagtttgtggcgatgctt-3′
vGlut	5′- gtcatgactatcatcgtacccatc-3′	5′- gtagcttccatcccgaaacc-3′
actg1	5′- tagttcatgtggctcggtca-3′	5′- gctggggactgactgacttt -3′
eef1	5′-tgtggtggaatcgacaaaag -3′	5′-cccaggcatacttgaaggag -3′

Gene expression was analyzed with qBase 1.3.5 software using the comparative cycle threshold method, yielding [delta][delta]C_t_ = [delta]C_t,sample −_ [delta]C_t,reference_.

### Quantification of Plasma Corticosterone Levels

Plasma samples (dilution 1/40) were assayed by a commercially available enzyme-linked immunoabsorbent assay (ELISA, Corticosterone EIA Kit, Enzo Life sciences, Switzerland). The intra-assay coefficient of variation was 8% and 6.6% for low and high concentrations, respectively. The inter-assay coefficient of variation was 13.1% and 7.8% for low and high concentrations, respectively.

### Statistics

All results are shown as mean+SEM and were analyzed using the software SPSS 17.0. Comparisons of the two groups were done using independent t-tests, whereas repeated measures analysis of variance (ANOVA) was used for comparisons of stimulus preferences (sociability, social memory, bedding preference; stimulus as within-subjects factor, virus as between-subjects factor). Time-dependent behavioral parameters were analyzed using 2-way repeated measures analysis of variance (ANOVA). Thereby, virus was taken as between-subjects factor and time as a within-subjects factor. In all analyses, the level of statistical significance was set at P<0.05.

## Results

### Nlgn2-overexpression in the Dorsal Hippocampus

**Figure 1:**
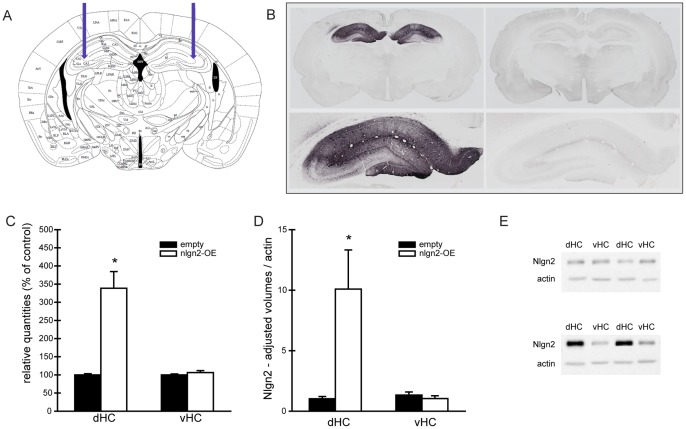
Verification of viral nlgn2-overexpression. A, Targeted area in the hippocampus for virus injections. **B, DAB immunohistochemistry** for nlgn2 in coronal sections from nlgn2-OE (left column) and empty (right column) with 20× magnification of the hippocampus (bottom panel). **C, Relative quantities of nlgn2** measured in dorsal (dHC) and ventral hippocampus (vHC) (dHC: T_(4.038)_ = −5.193, p<0.01; vHC: T_(9)_ = −1.117, p>0.05). **D–E Western blot analysis of synaptoneurosome. D,** Nlgn2-OE rats showed significantly increased levels of nlgn2 in the synaptoneurosome compared to empty virus controls concerning the dorsal hippocampus (T_(4.027)_ = −2.797, p<0.05), but not in the ventral parts of the hippocampus (T_(9)_ = 0.836, p>0.05). **E,** representative western blots from synaptoneurosomes of the dorsal (dHC) and the ventral hippocampus (vHC) of empty virus controls (upper panel) and nlgn2-OE animals (bottom panel). Empty n = 6, nlgn2-OE n = 5. ns = Not significant, *significantly different from empty animals.

The adeno-associated virus overexpressing nlgn2 or containing an empty construct was injected in the stratum radiatum of the dorsal hippocampus as depicted in Fig. 1A & 1B. Quantification of mRNA levels by qPCR and protein levels by western blot validated the overexpression in the dorsal hippocampus of nlgn2-OE rats without significant spreading to the ventral hippocampus (Fig. 1C–E). DAB immunostaining confirmed the location of the overexpression of nlgn2 in the dorsal hippocampus of nlgn2-OE animals compared to animals injected with an empty virus (Fig. 1B). One animal injected with the nlgn2-overexpressing virus was excluded from all results due to lack of successful nlgn2-overexpression in the hippocampus as shown by qPCR analysis.

### Hippocampal Nlgn2-overexpression does not Affect Body Weight Gain

We did not observe differences in body weight between the groups throughout the experiments. In all groups, there was a gain in body weight over time, but the injection of nlgn2-OE virus did not affect this development as compared to empty virus animals (Suppl. Fig. S2).

### Hippocampal Nlgn2-overexpression Reduces Novel Object Exploration

**Figure 2:**
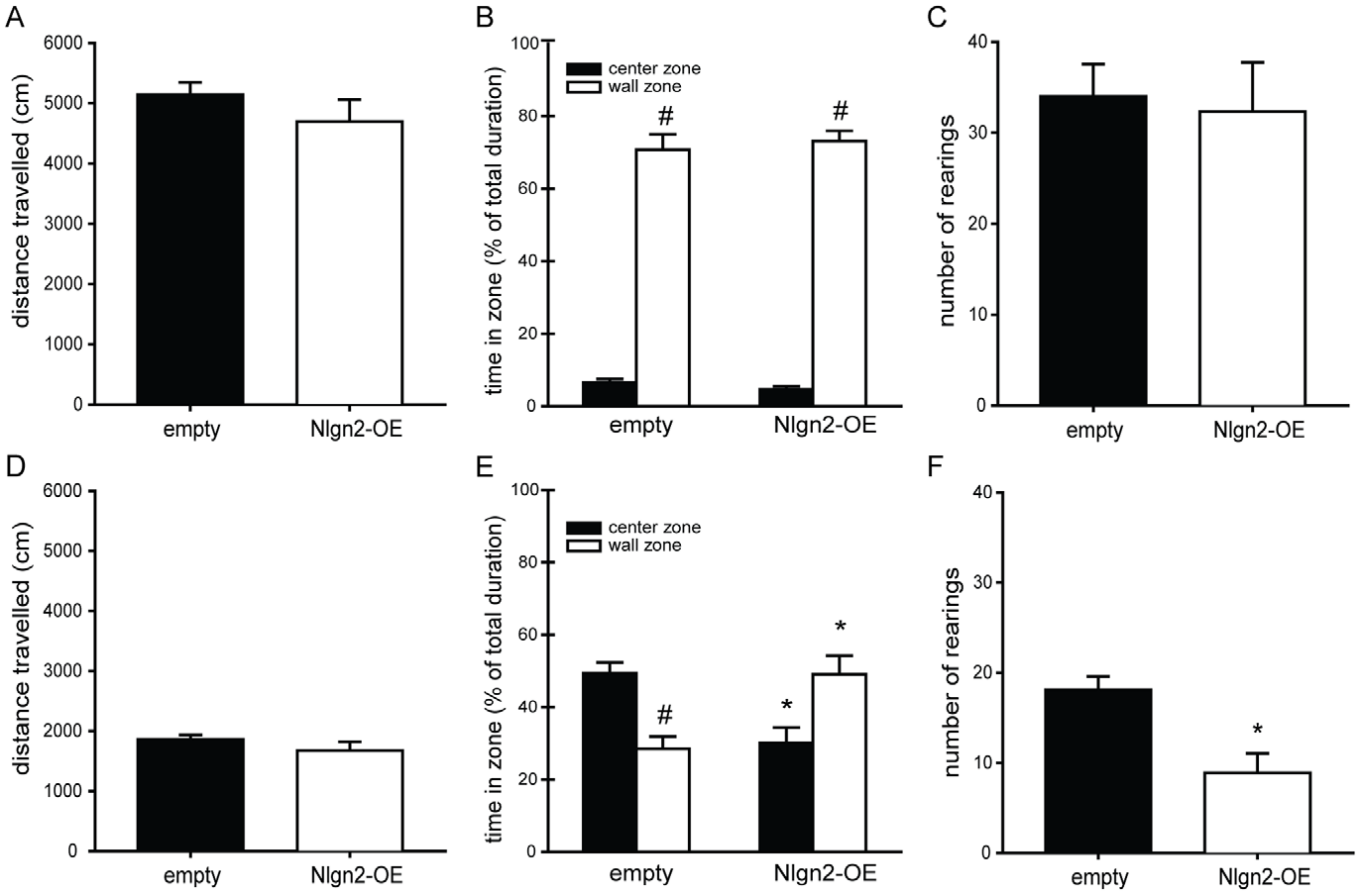
Open field-Novel object. A–C, Open field. A, Distance travelled in the open-field apparatus. No differences were observed concerning exploration between the groups (empty 5141.94±202.83, nlgn2-OE 4694.12±363.56, t-test ns). **B,** Time spent in zones of the open-field. All animals spent more time in the wall zone than in the center zone (paired t-test, empty p<0.001, nlgn2-OE p<0.001), but no difference between the groups could be found (F_(1,17_) = 0.461, p = 0.506). **C,** Number of rearings. No differences between the groups (empty 34.0±3.56, nlgn2-OE 32.3±5.41, t-test ns). **D–F Novel object. D,** Distance travelled during the novel object trial. The groups did not differ in terms of travelled distance (empty 1864.98±72.31, nlgn2-OE 1676.04±147.7, t-test ns). **E,** Time spent in the zones during the NO trial. Empty virus control animals spent more time in the center exploring the object than in the wall zone (paired t-test, p<0.01), whereas nlgn2-OE animals spent significantly less time in the center compared to empty virus animals (empty 49.41±2.86, nlgn2-OE 30.03±4.35, p<0.001) and significantly more time in the wall zone compared to empty virus animals (empty 28.44±3.47, nlgn2-OE 49.06±5.18, p<0.01). Nlgn2-OE rats showed a strong trend to spend more time in the wall zone compared to the center zone, but this effect did not reach significance (paired t-test, p = 0.055). **F,** Number of rearings. Animals injected with the empty virus showed significantly more rearing during the NO trial compared to nlgn2-OE animals (empty 18.1±1.49, nlgn2-OE 8.9±2.16, p<0.01). Empty n = 10, Nlgn2-OE n = 9. ns = Not significant. *significantly different from empty animals, ^#^significantly different from center zone.

To investigate any influence of the viral overexpression of nlgn2 on locomotion and exploratory behavior, rats were subjected to the OF-NO test. We did not observe differences between animals injected with the empty virus and the overexpressing construct in terms of locomotor activity in the OF and NO trial (Fig. 2A&D). The time spent in the center compartment of the open field is generally considered as an aspect of anxiety-like behavior. Here, we didn’t see any differences between the animals in the OF test (Fig. 2B). All of them spent significantly more time in the wall zone compared to the center zone. Concerning the number of rearings, we did not see differences between the groups (Fig. 2C).

When a novel object was introduced to the center of the apparatus, nlgn2-OE rats spent significantly less time exploring the center containing the novel object compared to animals injected with the empty virus (Fig. 2E). Additionally, they spend more time in the wall zone compared to the center zone with the newly introduced object (Fig. 2E). We observed a significantly decreased number of rearings in nlgn2-OE animals when compared to animals injected with an empty construct at this stage of the test (Fig. 2F).

### Hippocampal Nlgn2-overexpression has no Impact on Sociability and Memory

**Figure 3:**
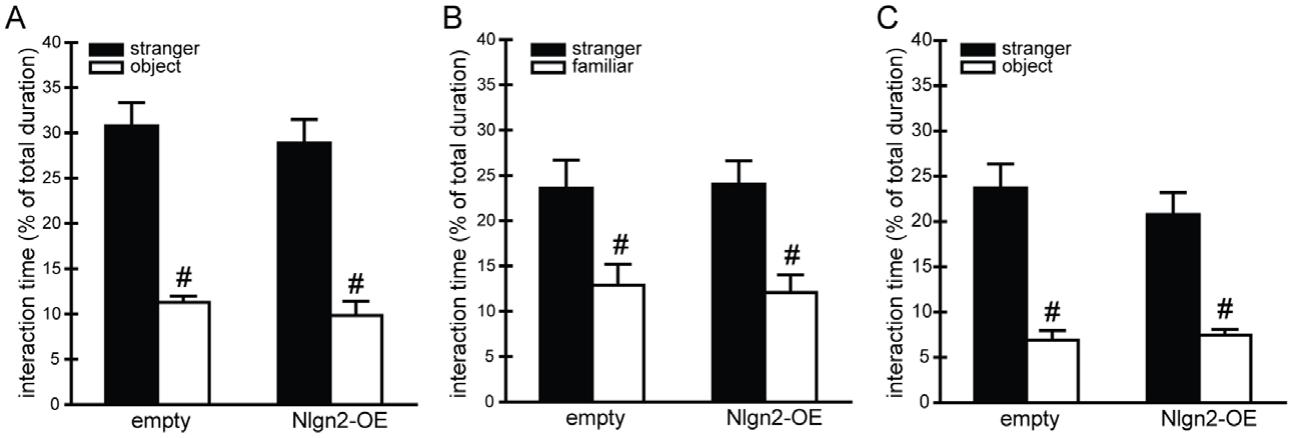
Social behavior. A–B Sociability-social memory. A, Sociability. Interaction times with stranger juvenile and object. All animals spent more time investigating the juvenile compared to the object (F_(1,18)_ = 65.40, p<0.001), but there was no difference between the groups. **B, Social memory.** Interaction times with stranger and familiar juveniles in the social memory trial. Both groups significantly explored more the stranger juvenile compared to the familiar juvenile (F_(1,18)_ = 19.614, p<0.001), which they already encountered in the first trial for the sociability test. No differences concerning the interaction times between the groups could be detected. **C,** **Sociability after RI test.** The animals of both groups spent significantly more time with the juvenile compared to the object (F_(1,18)_ = 58.023, p<0.001), but between the groups we did not observe any differences. Empty n = 10, Nlgn2-OE n = 9. ^#^significantly different from stranger interaction time.

Animals were subjected to the combined test for sociability and social memory. All subjects spent more time exploring the juvenile compared to the object in the sociability test (Fig. 3A). Regarding the social memory trial, all animals spent more time sniffing at the wire cage containing the stranger juvenile compared to the already familiar one (Fig. 3B).

After cohabitation with females and the aggressive encounter in the resident-intruder test, we tested all animals again for sociability. Animals of both groups spent more time investigating the juvenile compared to the object, but no differences between the groups could be seen (Fig. 3C).

### Hippocampal Nlgn2-overexpression Inhibits Aggression

**Figure 4:**
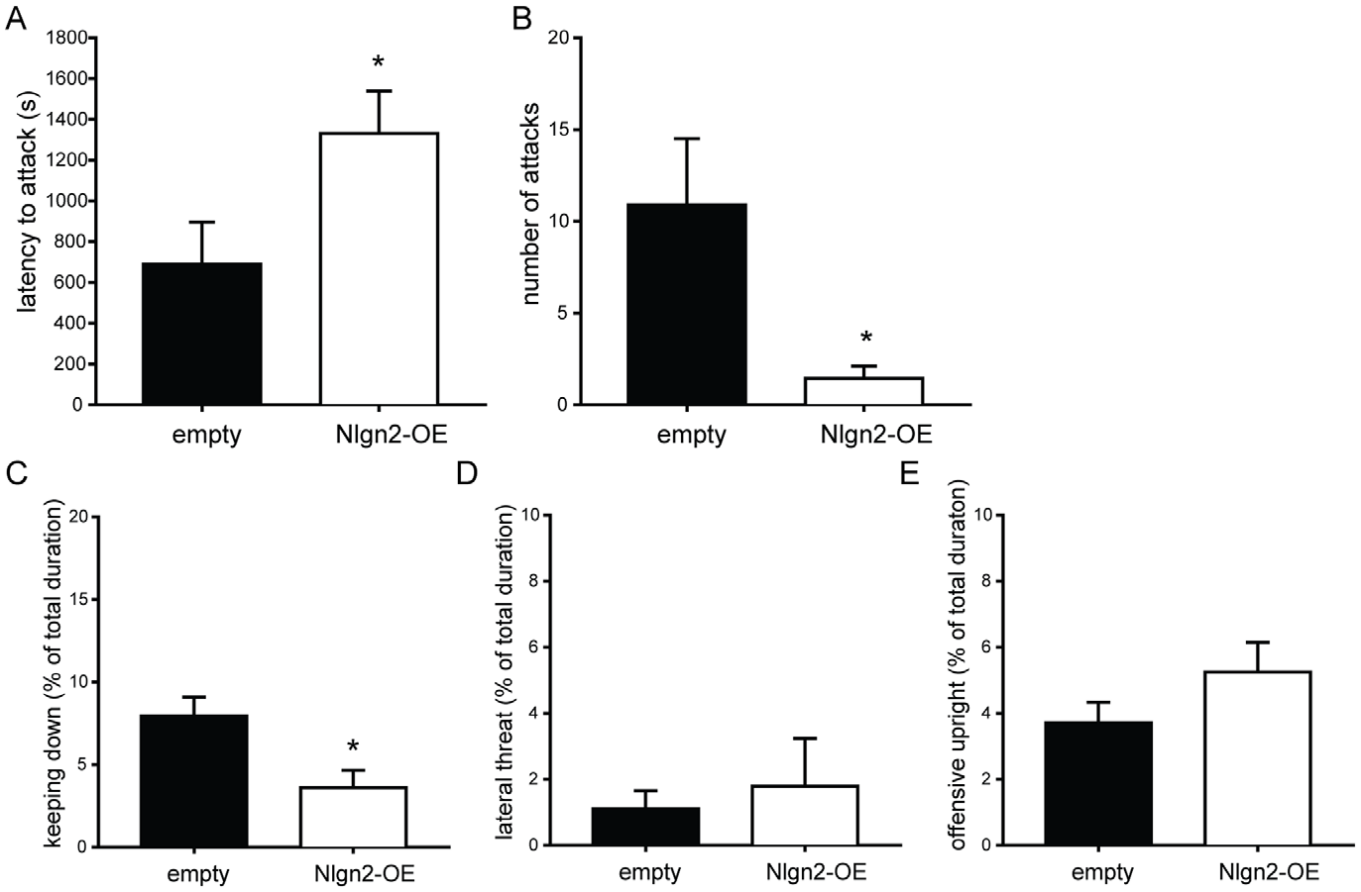
Aggression-related behavior. A, Latency to attack. In line with the number of attacks, nlgn2-OE animals had significantly longer latencies to attack the unfamiliar intruder than empty animals (empty 689.0±206.73, nlgn2-OE 1332.06±208.10, p<0.05). **B, Number of attacks during RI.** Animals injected with nlgn2-OE virus showed significantly less attacks towards the intruder compared to control animals (empty 10.9±3.62, nlgn2-OE 1.44±0.67, p<0.05). **C, Keeping down behavior.** Rats with the nlgn2-OE construct showed significantly less keeping down behavior towards the intruder compared to controls (empty 143.26±20.08, nlgn2-OE 64.89±18.90, p<0.05). **D–E, Lateral threat (D) and offensive upright (E).** No differences could be observed concerning the aggression-related behaviors lateral threat (empty 19.91±9.79, nlgn2-OE 32.16±26.08, t-test ns) and offensive upright posture (empty 66.80±11.11, nlgn2-OE 94.51±16.08, t-test ns). Empty n = 10, Nlgn2-OE n = 9. ns = Not significant. *significantly different from empty animals.

In the resident-intruder test, the latency to attack the intruder was significantly longer in nlgn2-OE animals compared to control animals (Fig. 4A). Moreover, animals injected with the nlgn2-overexpressing virus attacked the intruder less often compared to animals injected with the empty construct (Fig. 4B). Besides the actual attacks, we also scored 3 aggression-related types of behavior: keeping down, lateral threat and offensive upright. For keeping down behavior, the strongest parameter for offensive behavior, we could observe that nlgn2-OE animals spent less time keeping down the intruder compared to controls with the empty virus (Fig. 4C). In lateral threat and offensive upright, there were no differences between the groups (Fig. 4D & 4E).

We did not find differences in corticosterone levels in the plasma samples taken 0 and 30 minutes after the RI test (data not shown).

### Hippocampal Nlgn2-overexpression does not Affect Olfaction

All animals were tested for olfaction in order to exclude any alterations of olfaction being responsible for behavioral abnormalities. All rats preferred the male bedding compared to the clean bedding without any differences between the two experimental groups (data not shown).

### Hippocampal Nlgn2-overexpression Results in Altered Gene Expression

**Figure 5:**
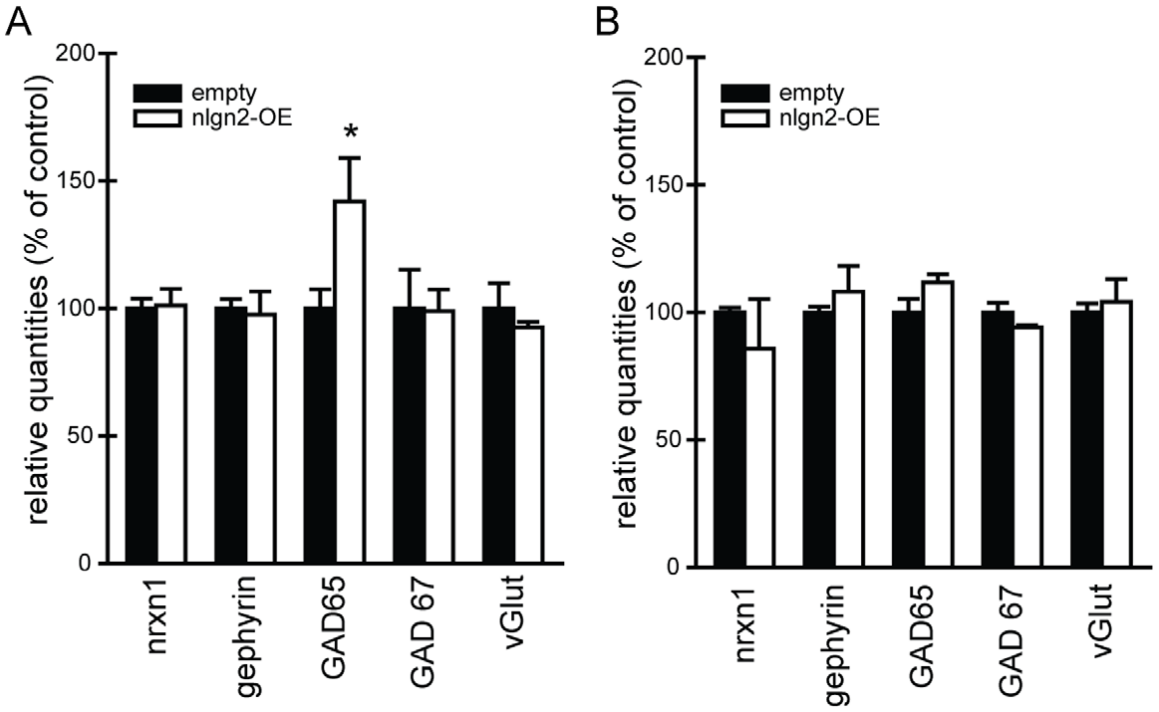
mRNA expression. A, mRNA expression in the dorsal hippocampus. Nrxn1: T_(9)_ = −0.155, p>0.05; Gephyrin: T_(9)_ = 0.279, p>0.05; GAD65: T_(9)_ = −2.404, p<0.05*; GAD67: T_(9)_ = 0.059, p>0.05; vGlut: T_(5.439)_ = 0.739, p>0.05. **B, mRNA expression in the ventral hippocampus.** Nrxn1: T_(4.071)_ = 0.732, p>0.05; Gephyrin: T_(9)_ = −0.850, p>0.05; GAD65: T_(9)_ = 0.380, p>0.05; GAD67: T_(9)_ = 1.392, p>0.05; vGlut: T_(9)_ = −0.457, p>0.05. Empty n = 6, nlgn2-OE n = 5. *significantly different from empty animals.

After behavioral testing, animals (6 of each group) were sacrificed under basal conditions and brain halves were taken to investigate mRNA expression levels in the dorsal and ventral hippocampus by qPCR (Fig. 5A & 5B, respectively). We did not observe any differences between the groups concerning the expression levels of Neurexin1 (nrxn1), the presynaptic binding partner for nlgn2, and gephyrin, a postsynaptic interaction partner of nlgn2 in inhibitory synapses. We checked for two forms of the glutamate decarboxylase, GAD65 and GAD67, as markers for GABAergic synapses. We found an increase of GAD65 in the dorsal hippocampus in nlgn2-OE animals compared to animals injected with the empty virus, but GAD67 was not differentially expressed. Regarding vGlut as marker for glutamatergic nerve terminals, we did not find differential expression levels of the two groups. In the ventral hippocampus, no gene expression differences were detected.

## Discussion

In the present study, we evaluated the impact of overexpressing nlgn2 specifically in the dorsal hippocampus of adult rats using an adeno-associated virus. We found altered behavior in terms of reduced novelty-induced exploration and decreased offensive behaviour, whereas sociability and social memory remained unchanged. Additionally, we found GAD65 expression levels to be increased specifically in the dorsal hippocampus of nlgn2-OE animals. Expression of GAD67, or other nlgn2-interacting partners and the excitatory marker vGlut explored, did not differ between groups.

In the OF test, rats overexpressing nlgn2 revealed normal locomotor activity and anxiety-related behavior, but in the novel object trial they spent significantly less time exploring the novel stimuli compared to control animals and additionally showed thigmotaxis as demonstrated by the higher percentage of time spent in the wall zone. Consistently, mice with conditional nlgn2-overexpression showed thigmotaxis in the open field test, while the distance travelled throughout the arena was unchanged [25]. Additionally, the overexpression of nlgn2 led to a reduced number of rearings in the novel object trial, but not in the open field, which is in line with the reduced time exploring the novel object. In contrast, animals with the conditional overexpression of nlgn2 showed more rearings in the OF task [25], but since the nlgn2-OE is not restricted to the hippocampus and occurs throughout development, this effect might be due to compensatory mechanisms or changes in nlgn2 levels in other brain regions. Rearing behavior is considered as a parameter for exploration, mostly in response to novel stimuli [37], meaning that the nlgn2-OE decreased exploratory behavior both in terms of time spent exploring a novel object and rearing behavior. The hippocampus has been shown to be associated with exploratory behavior [37], [38], rendering the specific manipulation of nlgn2 levels in the hippocampus most likely responsible for these behavioral changes. The observations mentioned are in line with behavioral alterations shown in animal models of autism and may be analogous to restricted interests in humans with autism [39], [40].

We did not find an effect of the overexpression of hippocampal nlgn2 on social interaction or social memory. A few studies showed an association of altered nlgn2 expression with social behavior [28] and manipulation of nlgn2 expression led to reduced social interactions [25]. However, deletion of nlgn2 did not have any effect on social behavior [41]. In our experiment, we used a very specific approach in terms of timing and location of the overexpression, which might not be comparable to longer lasting or more widespread manipulations of nlgn2.

Several lines of evidence show an involvement of the hippocampus in aggression-related behavior [42], [43], which is another aspect of social behavior implicated in autism and schizophrenia [44], [45]. We found reduced aggressive-like behavior in rats with viral nlgn2-overexpression. Especially, the number of attacks was reduced, while the latency to attack was increased. Animals with nlgn2-OE showed decreased time of keeping down the intruder, which represents one of the strongest parameters for offensive behavior. This is, to our knowledge, the first demonstration of the involvement of nlgn2 in the hippocampus in aggression-related behavior. Further studies with manipulated levels of nlgn2 like a viral knock-down could confirm our results and the hypothesis of a modulating role of nlgn2 in the hippocampus.

Many studies so far focused on the prefrontal cortex and its influence on social and emotional behavior [46], [47]. The implication of the hippocampus in social behavior and aggression is relatively new, but in support of previous data from our laboratory [28], [48] and the literature [42], [43], [49], [50], [51]. Yet, it is unclear how a shift in the balance of the E/I ratio impacts on altered aggressive behavior, but there is evidence linking enhanced GABAergic transmission with reduced aggressive-like behavior [52]. Reduced aggression-related behavior after shifting the E/I ratio towards inhibition in the hippocampus by nlgn2-OE is in line with these observations and might render the hippocampus as potential candidate to exert inhibitory effects on aggression.

On the molecular level, overexpression of nlgn2 in the hippocampus was found to lead specifically to altered expression of inhibitory synapse-associated molecules. In detail, we found increased levels of GAD65, but not GAD67. These two isoforms of GAD, which catalyze the decarboxylation of glutamate to GABA, derive from two independently regulated genes [53]. GAD65 preferentially synthesizes GABA in the synaptic terminal for vesicle release, whereas GAD67 preferentially synthesizes cytoplasmic GABA [54]. We did not observe changes in excitatory synaptic markers as vGlut expression was not altered by nlgn2-overexpression. Regarding the enrichment of nlgn2 specifically at inhibitory synapses and its association with the modulation of inhibitory synapse function [8], [7], [5], [55], [10], [56], our results indicate an increase of inhibitory synaptic transmission by means of enhanced vesicle GABA release after nlgn2-overexpression. In line with our findings, nlgn2-OE *in vitro* was shown to increase inhibition and nlgn2-knockout *in vitro* and *in vivo* resulted in decreased inhibitory synaptic responses with no effect on excitatory responses [41], [2]. Additionally, paired recordings in primary somatosensory cortex in mice lacking nlgn2 revealed decreased inhibitory postsynaptic current amplitudes in single fast-spiking interneurons but not in single somatostatin-positive inhibitory interneurons, suggesting nlgn2 not being necessary for establishing unitary inhibitory synaptic connections but being selectively required for “scaling up” those connections [57]. Further, conditional overexpression of nlgn2 in the forebrain of mice also resulted in increased levels of VGAT, a marker for inhibitory synapses, together with augmented vGlut levels [25]. The latter might be a compensatory effect over time for the increased inhibitory synaptic transmission caused by enhanced nlgn2 levels.

As we did not find changes in the expression of the nlgn2-interacting molecule gephyrin on the postsynaptic side and neurexin1 in the presynapse, the precise modulatory effect of inhibitory synaptic transmission might be specifically mediated by nlgn2. Modulation of nlgn2 levels may affect only selected pre- and postsynaptic structural features and mechanisms, whereas the precise effects of altered nlgn2 levels on its linked pathways remain unclear [27], [58], [59], [60].

An imbalance of excitation and inhibition in the brain is implicated in several psychiatric disorders as possible cause for behavioral alterations [9], [14], [15], [16]. Conditional nlgn2-OE mice showed disturbances of this E/I balance in the brain [25]. Our results might represent a similar shift towards inhibition in terms of increased GAD65 but unchanged vGlut.

Regarding the dynamics of nlgn2 expression levels during early life stages [7], nlgn2 might possibly be involved in the development of social and emotional behavior. Therefore, further investigation in young animals might elicit the effect of altered nlgn2 expression during early life on social behavior. Further, given the close functional interaction of the hippocampus with the amygdala, and the known important role of the amygdala in responses to novel objects and contexts as well as aggression and violence [61], [62], it may be possible that some of the observed behavioral effects are mediated, at least in part, via the amygdala.

Taken together, rats with an overexpression of nlgn2 in the hippocampus show increased inhibition in novelty-induced exploration and reduced aggressive behavior. Alterations in aggression are implicated in several diseases like schizophrenia, depression and autism. A shift of the E/I balance in the brain was proposed as an underlying mechanism for those diseases. Indeed, the results of our study might be in line with this notion of a shift towards inhibition as animals with nlgn2-overexpression also showed increased GABA-synthesizing GAD65 in nerve terminals, whereas vGlut as marker for excitatory synapses and nlgn2-interaction partners in pre- and postsynapse, gephyrin and neurexin1 respectively, remained unchanged. Our data add to the growing evidence of a hippocampal involvement in social behavior and suggest nlgn2 as a potential target for treatment interventions.

## Supporting Information

Figure S1 **Timeline of the experiment.** Animals were kept together with the dam until weaning on postnatal day 21. Afterwards, they were housed in groups of three under standard conditions. One week before surgery, anxiety levels were assessed in the EPM in order to balance the groups with regard to anxiety for AAV surgery. At the age of 12 weeks, animals underwent surgery with injection of either a nlgn2-overexpressing adeno-associated virus or an empty construct. After a recovery period of 5 weeks, all rats were screened in the open-field/novel object test. One week later, the combined sociability – social memory test was conducted and cohabitation with females started on the day after. On the 10th day of cohabitation, animals were subjected to the resident-intruder test with another sociability test three days later. On the 20th day, cohabitation was stopped. Following two weeks of single-housing, all rats were tested for olfaction and 6 days later, 4 animals were sacrificed by perfusion and 6 remaining animals were sacrificed by decapitation.
(TIF)

Figure S2 **Body weight.** We observed a time effect throughout the experiment (F_(5,13)_ = 107.803, p<0.001), but we did not find a time*virus interaction (F_(5,13)_ = 1.120, p>0.05). Animals injected with the nlgn2-OE virus did not differ from empty virus animals concerning body weight during the experiment. Empty n = 10, Nlgn2-OE n = 9.
(TIF)
