# Supplementary figures and images for: Hippocampal Neuroligin-2 Overexpression Leads to Reduced Aggression and Inhibited Novelty Reactivity in Rats
Source: PLoS One. 2013 Feb 22;8(2):e56871. doi: 10.1371/journal.pone.0056871 (PMC3579928; doi:10.1371/journal.pone.0056871)

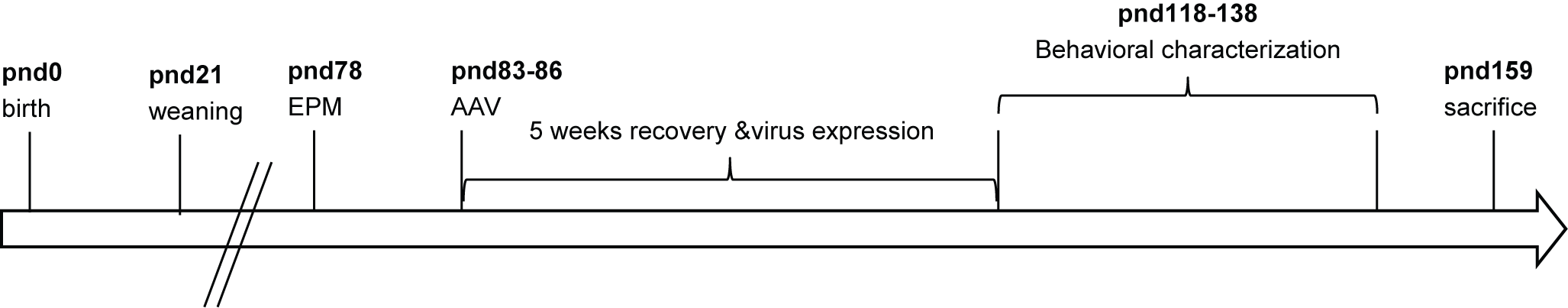

Supplement: Figure S1 — Timeline of the experiment. Animals were kept together with the dam until weaning on postnatal day 21. Afterwards, they were housed in groups of three under standard conditions. One week before surgery, anxiety levels were assessed in the EPM in order to balance the groups with regard to anxiety for AAV surgery. At the age of 12 weeks, animals underwent surgery with injection of either a nlgn2-overexpressing adeno-associated virus or an empty construct. After a recovery period of 5 weeks, all rats were screened in the open-field/novel object test. One week later, the combined sociability – social memory test was conducted and cohabitation with females started on the day after. On the 10th day of cohabitation, animals were subjected to the resident-intruder test with another sociability test three days later. On the 20th day, cohabitation was stopped. Following two weeks of single-housing, all rats were tested for olfaction and 6 days later, 4 animals were sacrificed by perfusion and 6 remaining animals were sacrificed by decapitation. (TIF) [file pone.0056871.s001.tif]

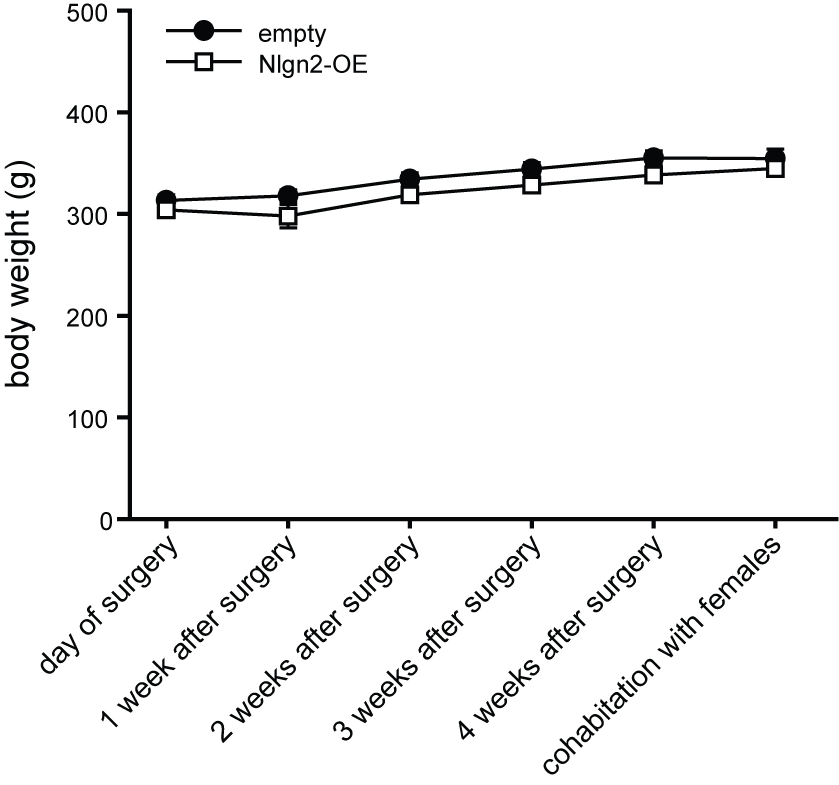

Supplement: Figure S2 — Body weight. We observed a time effect throughout the experiment (F(5,13) = 107.803, p<0.001), but we did not find a time*virus interaction (F(5,13) = 1.120, p>0.05). Animals injected with the nlgn2-OE virus did not differ from empty virus animals concerning body weight during the experiment. Empty n = 10, Nlgn2-OE n = 9. (TIF) [file pone.0056871.s002.tif]
